# Adolescent Ethanol Exposure Leads to Stimulus-Specific Changes in Cytokine Reactivity and Hypothalamic-Pituitary-Adrenal Axis Sensitivity in Adulthood

**DOI:** 10.3389/fnbeh.2017.00078

**Published:** 2017-05-04

**Authors:** Andrew S. Vore, Tamara Doremus-Fitzwater, Anny Gano, Terrence Deak

**Affiliations:** ^1^Behavioral Neuroscience Program, Department of Psychology, Binghamton UniversityBinghamton, NY, USA; ^2^Department of Psychology, Ithaca CollegeIthaca, NY, USA

**Keywords:** adolescent intermittent ethanol, rat, sex differences, HPA axis, cytokine

## Abstract

Adolescent alcohol use comprises a significant public health concern and is often characterized by binge-like consumption patterns. While ethanol exposure in adulthood has been shown to alter the stress response, including the Hypothalamic–Pituitary–Adrenal (HPA) axis, few studies have examined whether binge-like ethanol exposure during adolescence results in enduring changes in HPA axis sensitivity in adulthood. In the present studies, adolescent Sprague-Dawley rats were given intragastric (i.g.) intubations of ethanol (4 g/kg) or vehicle once per day for three consecutive days, beginning on postnatal day (P) 30 (±1). This exposure was followed by a 2-day period of rest/withdrawal. Rats received a total of either two (Experiments 1, 2 and 3) or four (Experiment 4) cycles of ethanol exposure and were subsequently allowed to age normally until adulthood. In Experiment 1, adult, (P71–75), ethanol- or vehicle-exposed rats received a 60 min restraint stress challenge. In Experiment 2, rats received a 50 μg/kg injection of lipopolysaccharide (LPS). In Experiment 3, rats received a challenge of 2.5 g/kg ethanol (intraperitoneally; i.p.). In Experiment 4, male and female ethanol- or vehicle- exposed rats received a 50 μg/kg injection of LPS. In all experiments, blood samples were collected for later assessment of corticosterone (CORT), blood ethanol concentrations (BECs), and the cellular fraction of blood was analyzed for cytokine gene expression. As expected, all three challenges led to a time-dependent surge in CORT. Gene expression analyses of cytokines (Interleukin [IL]-6, IL-1β, and Tumor necrosis factor alpha [TNFα]) from the cellular fraction of blood revealed unique, time-dependent patterns of cytokine expression depending upon the nature of the adult challenge incurred (restraint, LPS, or EtOH). Importantly, adolescent ethanol exposure led to attenuated restraint and LPS-induced cytokine expression in males, whereas female rats displayed an absence of cytokine alterations, and a tendency toward heightened HPA axis reactivity. These findings suggest that adolescent ethanol exposure may cause lasting alterations in cytokine regulation and HPA axis sensitivity that (a) persist into adulthood; (b) may vary depending on the nature of the challenge incurred during adulthood; and that (c) are sex-specific.

## Introduction

Alcohol use and abuse disorders comprise a substantial public health and financial burden, resulting in an estimated 3.3 million deaths per year globally and $223.5 billion in financial burden to the United States alone (Centers for Disease Control and Prevention, 2014; World Health Organization, 2014). It has been estimated that almost 75% of this impact can be attributed to binge drinking, defined as ethanol consumption that results in Blood Ethanol Concentrations (BECs) of 0.08 mg/dL or higher (Centers for Disease Control and Prevention, 2014). Alcohol consumption is prevalent in adolescence and a large percentage of adolescent individuals that consume alcohol exhibit binge-like consumption patterns. These patterns of intake typically begin in early to late adolescence, peaking in early adulthood (21–25 years of age), with consumption tapering across adulthood in individuals that exhibit normative alcohol use. Binge-like alcohol consumption can be particularly harmful, and early, high frequency binge drinking has been shown to correlate with later alcohol use disorder (AUD) development (Grant and Dawson, 1997; Crews et al., 2016).

Adolescence is a time of unique sensitivity to the consequences of ethanol exposure. Mirroring what is seen in humans, adolescent rodents consume notably higher quantities of ethanol than adult counterparts (Doremus et al., 2005; Spear, 2016). Adolescent rats show altered reactivity to ethanol exposure with increased sensitivity to the positive effects of ethanol exposure and decreased sensitivity to the negative effects (Doremus et al., 2003; Varlinskaya and Spear, 2004a). Adolescent animals have also exhibited increased locomotor sensitization to ethanol and higher sensitivity to the motivational effects of ethanol (Pautassi et al., 2008) compared to adult counterparts that received similar quantities of ethanol. This differential reaction to a spectrum of consequences of ethanol exposure likely contributes to adolescent animals consuming higher quantities of ethanol, since insensitivity to negative consequences of high dose ethanol use (sedation, motor impairment, aversion) and hypersensitivity to various positive elements would seem to promote future ethanol consumption (Varlinskaya and Spear, 2004b).

A more concerning element of adolescent ethanol exposure is its ability to cause potentially lifelong changes in development. Adolescence is a critical time of neural refinement during which the brain undergoes a series of structural and functional changes. Increases in myelination, decreases in gray matter volumes, and a reduced total number of synapses indicate that adolescence is a time during which novel connections are being made and prior connections are being refined (Giedd, 2008). Underlying neurodevelopmental changes during adolescence may confer added potential for insult from substances such as ethanol (Guerri and Pascual, 2010). A common functional consequence of adolescent ethanol exposure is the retention of an adolescent-like phenotype into adulthood. This “locking in” of the adolescent phenotype into adulthood has been shown to occur in a variety of phenomena and is not seen in adult rats following exposure to similar quantities of ethanol. Behavioral phenomenon such as resilience to withdrawal-induced anxiety (Doremus et al., 2003), reduced sensitivity to the sedative effects of ethanol (Matthews et al., 2008), as well as reduced sensitivity to conditioned taste aversion (CTA) induction (Diaz-Granados and Graham, 2007) have all demonstrated this locking in-like effect following adolescent ethanol exposure when challenged during adulthood. This phenomenon has also been demonstrated to extend beyond just ethanol challenge effects. Observed baseline differences in impulsivity, novelty seeking, and many other phenomena have also shown to linger into adulthood following adolescent ethanol exposure (see Spear and Swartzwelder, 2014 for a recent review).

Ethanol alone is a potent stimulator of the hypothalamic-pituitary-adrenal (HPA) axis as demonstrated by dose-dependent increases in plasma adrenocorticotropic hormone (ACTH) and corticosterone (CORT; Rivier et al., 1984; Doremus-Fitzwater et al., 2014). Importantly, cross sectional studies comparing adolescent and adult rats have indicated that stressors such as hypoxia and restraint stress result in prolonged ACTH and CORT responses when compared to adult rodents (Romeo et al., 2016), though differences in peak CORT responses were not observed. A similar difference occurs following ethanol challenge as well, where adolescents exhibit delayed recovery (or shutoff) of the axis relative to adults. While this effect was more pronounced in female rats, both male and female adolescent rats have shown increased CORT at several points after ethanol when compared to adult animals challenged with the same dose of ethanol (Willey et al., 2012). In contrast, prior exposure to ethanol in adolescence has been shown to blunt the HPA-axis response to subsequent ethanol exposure up to 24 days later (Lee and Rivier, 2003). Thus, intrinsic differences in the HPA axis response to stress between adolescents and adults are now well-precedented in the literature, though the mechanisms underlying these differences remain obscure.

Beyond effects on the HPA axis, ethanol also has substantial effects on cytokine release and the immune system. Cytokines are a class of proteins that play a key role in propagation of the inflammatory response evoked by immunological threats, tissue damage, and other challenges such as stress (see Deak et al., 2015 for a recent review). In particular, the pro-inflammatory cytokines IL-1, TNF-α and IL-6 are rapidly induced by immune challenges or psychological stress, and often act synergistically with one another to influence host defense and tissue repair. These cytokines also potently induce HPA axis activation, with glucocorticoids in turn influencing subsequent cytokine expression and activity. In this way, HPA-immune interactions serve as an exemplar of bi-directional communication between the brain and the immune system.

From a longitudinal perspective, recent studies have begun to explore whether the adolescent period also represents a critical period during which ethanol exposure can produce life-long changes in HPA axis sensitivity and immune function. For instance, adolescent ethanol exposure (6 exposures to 3 g/kg intraperitoneal (i.p.)) in male rats resulted in reduced baseline CORT and sensitized CORT release following acute or repeated ethanol challenge in adulthood (Przybycien-Szymanska et al., 2010, 2011). These changes in CORT patterns correlated with decreased vasopressin (AVP) and increased corticotropin-releasing hormone (CRH) within the paraventricular nucleus of the hypothalamus (PVN) of adolescent ethanol exposed rats following acute and binge ethanol challenge, suggesting alterations in central regulation of HPA axis regulation. In contrast, a more recent study showed that adolescent ethanol exposure (daily exposure to ethanol vapor for 6 h maintaining blood alcohol levels, BALs of 200 mg/dL) blunted the HPA axis response to adult ethanol challenge (3.2–4.5 g/kg intragastric (i.g.); Allen et al., 2016). Though dose, route and frequency of ethanol exposure during adolescence are likely explanations for the differences between these studies, a more fundamental question regarding the potential for stressor-specific outcomes in adults with a history of adolescent ethanol exposure still remains.

With this in mind, our primary goal was to establish how binge-like ethanol exposure during adolescence would alter cytokine and HPA axis responses to stress challenges incurred during adulthood. In doing so, we adopted an adolescent alcohol exposure procedure that has previously been shown to produce changes lasting to adulthood in HPA axis regulation, including alterations in CORT release as well as changes in AVP and CRH following binge and acute ethanol exposure (Przybycien-Szymanska et al., 2010, 2011). Following intermittent binge ethanol exposure in adolescence, three distinct studies were executed in which rats were given different challenges in adulthood to test whether axis sensitivity may depend upon the nature/modality of the stress challenge experienced as adults. Thus, adult rats with a history of adolescent ethanol exposure were challenged by restraint, lipopolysaccharide (LPS) administration, or adult ethanol exposure (Experiments 1–3 respectively). Though our primary interest was HPA axis sensitivity indicated by plasma CORT responses, we also had a secondary interest in how adolescent intermittent ethanol (AIE) would influence the expression of cytokines within the cellular fraction of whole blood as a secondary index of stress sensitivity, and due to the potential mechanistic role that plasma cytokines might play in HPA axis responses (Rivest et al., 2000; Szelényi, 2001). In human research, analysis of peripheral cytokine levels has been shown to be useful in monitoring general immune activation and to correlate well with expected levels of neuroimmune activation (Sullivan et al., 2006). In addition, elevated levels of peripheral pro-inflammatory cytokines have been shown to correlate with psychiatric illness in a specific subset of individuals (Fillman et al., 2016). Finally, due to the literature demonstrating that male animals display profound HPA axis blunting following adolescent ethanol exposure, we examined potential sex differences in HPA axis reactivity following adolescent alcohol exposure.

## Materials and Methods

### General Methods

#### Subjects

In the first three studies, male Sprague-Dawley rats (*N* = 50) were purchased from Harlan Laboratories and shipped at postnatal day (P) 22 ± 1. In the fourth study, male and female Sprague-Dawley rats (*N* = 30) were bred in-house using breeders originally derived from Harlan (Envigo). With day of birth being deemed postnatal day (P) 0, rats were allowed to develop normally until P21 at which point animals were weaned and pair-housed with a same-sex partner from a different litter in standard Plexiglas bins. In all cases, rats were given 1 week to acclimate to colony conditions and were then handled (P29) for 2–3 min to acclimate them to human contact prior to experimentation. Colony conditions were maintained at 22 ± 1°C on a 12:12 light:dark cycle. Animals were pair-housed in standard Plexiglas bins with *ad libitum* access to food and water. Rats housed in pairs were always assigned to the same experimental group. In all experiments, rats were treated in accordance with Public Health Service (PHS) policy and all experimental protocols were approved by the Institutional Animal Care and Use Committee (IACUC) at Binghamton University.

#### Adolescent Alcohol Exposure Procedure

Starting at early adolescence (P30–32) rats received 4.0 g/kg intragastric (i.g.) intubations of ethanol or an equivolume dose of vehicle (tap water) once per day at approximately 1000 h. When administered i.g., ethanol solution was prepared daily using 95% ethanol stock diluted to a final working concentration of 20% in tap water. Weights were taken daily at least 90 min prior to intubations. Animals received three consecutive days of intubations followed by a 2-day period of rest/withdrawal during which animals remained undisturbed in their home cage. This cycle of intubations (3 days on, 2 days off) was then repeated a second time (Experiments 1–3), reaching its conclusion on P40–42. In Experiment 4, this cycle was administered four times, spanning ages of approximately P30–P50. Rats were then reared to adulthood without further manipulation until weights were again collected on P73–75. At this time, rats were randomly divided into cohorts for independent experiments.

#### Drug Preparations

When injected i.p., ethanol was prepared in a 20% solution mixed fresh daily using 95% ethanol stock with sterile physiological saline used as the vehicle. LPS (from serotype E0111:B4; Sigma Chemical Co.) solution was initially diluted 1.0 mg/mL using sterile (pyrogen-free) physiological saline and stored in frozen aliquots at −20°C until needed. On the day of experimentation, an aliquot of LPS was thawed and mixed fresh daily to the working concentration of 50 μg/mL LPS and delivered on a 1 mL/kg basis, also in pyrogen-free physiological saline.

#### Blood Sampling and Processing Procedure

In each experiment, rats were removed from their home cage and briefly placed into restraint tubes for the acquisition of blood samples. Blood samples were collected using the tail clip method in which the last 0.5–1.0 mm of the tail was transected. The tail was then stroked until whole blood was obtained as previously described (Hueston and Deak, 2014b). Rats were immediately returned to their home cage following each time point except in conditions where rats were assigned to the restraint stress condition. Animals were returned to their home cage within 2 min after first intrusion. Following collection, plasma was immediately separated through refrigerated centrifugation and stored at −20°C until analysis. RNA was extracted from the remaining fractionated layers of leukocytes and erythrocytes. Prior studies have shown that RNA extraction of whole blood is possible (Schwochow et al., 2012). Due to elimination of the plasma fraction and the lack of nuclei or cell organelles such as mitochondria in erythrocytes, it was reasoned that extraction on the combined buffy layer and erythrocyte fractions would largely reflect gene expression changes in leukocytes. The combined layers were stored at −80°C until analysis of expression of blood cytokine levels using Real Time Reverse Transcription Polymerase Chain Reaction (RT-PCR).

#### Corticosterone Measures

Quantitative determinations of plasma CORT levels were assessed using a CORT EIA kit (Cat No: ADI-901-097; Enzo Life Sciences, Farmingdale, NY, USA) as described in Hueston and Deak (2014a) with an inter-assay variability of 7.616% and an assay sensitivity of 27.0 pg/mL. Manufacturer’s instructions were followed for all steps except that samples were heat inactivated by immersion in 75°C water for a period of 60 min to denature endogenous CBG.

#### Reverse-Transcription Polymerase Chain Reaction

All RT-PCR was conducted using procedures described in previous work (Hueston and Deak, 2014a). Blood pellets were stored at −80°C until the time of RNA extraction. Total RNA in the sample was homogenized in Trizol RNA reagent (Invitrogen) using a TissueLyser and 5 mm stainless steel beads (Qiagen). Total RNA was then extracted through the use of RNeasy mini columns (Qiagen) following manufacturer’s instructions. RNA yield and purity were evaluated using a Nanodrop system (ThermoScientific) and cDNA was synthesized using a QuantiTect reverse transcription kit (Qiagen). All RT-PCR was run using a CFX384 real-time PCR detection system (Bio-Rad). All results were normalized to β-Actin as a housekeeper gene. Primer sequences for all targets run can be found in Table 1.

**Table 1:** **Primer sequences and accession numbers**.

Primer	Accession number	Oligo	Sequence
β-Actin	NM_031144.3	Forward	5′-GTCGTACCACTGGCATTGTG-3′
		Reverse	5′-GCCATCTCTTGCTCGAAGTC-3′
IL-6	NM_012589	Forward	5′-TAGTCCTTCCTACCCCAACTTCC-3′
		Reverse	5′-TTGGTCCTTAGCCACTCCTTC-3′
IL-1β	NM_031512	Forward	5′-AGGACCCAAGCACCTTCTTT-3′
		Reverse	5′-AGACAGCACGAGGCATTTTT-3′
TNF-α	NM_012675	Forward	5′-GGGGCCACCACGCTCTTCTG-3′
		Reverse	5′-CGACGTGGGCTACGGGTTG-3′
IκBα	NM_080899	Forward	5′-CTGTTGAAGTGTGGGGCTGA-3′
		Reverse	5′-AGGGCAACTCATCTTCCGTG-3′

#### Blood Ethanol Concentrations

BECs were determined from 5 μL samples of plasma using an Analox AM-1 alcohol analyzer (Analox Instruments, Lunenburg, MA, USA; as described in Doremus-Fitzwater et al., 2015). The machine was calibrated using a 200 mg/dL standard and tested every 15 samples using an alcohol quality control standard of known concentration purchased from Analox Instruments. Samples below 10–15 mg/dL were within a range considered to be noise and as such were interpreted as 0 values.

#### Statistical Analyses

All analyses of CORT levels, BECs and cytokine expression were analyzed using a mixed factorial ANOVA (*p* < 0.05). Fisher’s Least Significant Difference (*p* < 0.05) was used as a *post hoc* examination in instances where two way interactions were noted to identify loci of significant difference. Outliers were defined as data points that were more extreme than ±2 standard deviations from a given experimental group’s mean. If an outlier was identified within a given PCR reaction, it was excluded from that specific analysis but not across the remainder of the gene targets. Before analyzing individual cytokine data, β-Actin was examined to determine differences that may exist between groups in expression of this target gene of reference. All gene expression was reported quantified relative to expression of this reference gene.

### Experiment 1: Axis Reactivity to Restraint Stress Challenge Following Adolescent Ethanol Exposure

**Figure 1:**
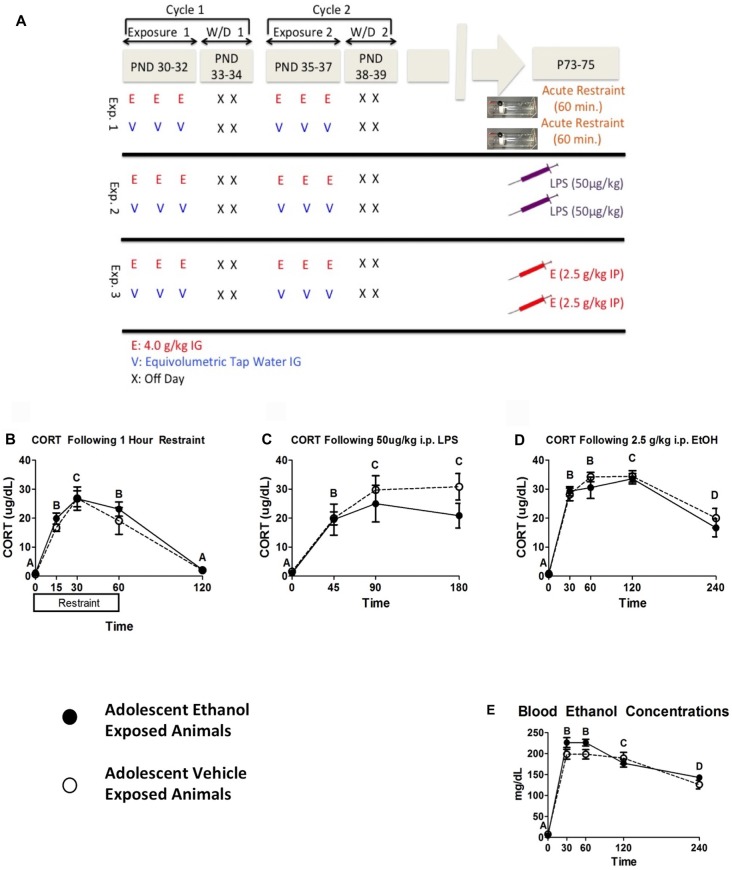
**Plasma Data from Experiments 1–3**. This figure shows a schematic diagramming Experiments 1–3 **(A)**. It also shows plasma corticosterone (CORT) levels following 1 h of restraint **(B)**, 50 μg/kg i.p. lipopolysaccharide (LPS) injection **(C)**, and 2.5 g/kg i.p. EtOH injection **(D)** and corresponding blood ethanol concentrations (BECs) in the ethanol challenge group **(E)**. Plasma was sampled 0, 15, 30, 60 and 120 min following restraint, 0, 45, 90 and 180 min following LPS challenge, and 0, 30, 60, 120 and 240 min following ethanol challenge. A significant main effect of time point is denoted by a lettering system in which time points that differ in letter are significantly different.

In the first experiment, restraint stress was chosen as a challenge due to its ability to reliably produce activation of the HPA axis and for reported differences between adolescent and adult CORT responses to restraint stress (Romeo et al., 2016). Male rats (*n* = 6–8 per group; *N* = 14) were given two cycles of intermittent ethanol exposure or vehicle intubations as described above. At adulthood (P73–75), rats from both groups were placed into clear Plexiglas restraint tubes and a baseline blood sample was collected immediately. Rats remained in restraint and subsequent blood samples were collected at 15, 30 and 60 min time points. Immediately after the 60 min time point, rats were returned to their home cage and a final recovery sample was collected 60 min later (i.e., 120 min following stress onset; see Figure 1A).

### Experiment 2: Axis Reactivity to Lipopolysaccharide Challenge Following Adolescent Ethanol Exposure

In the second experiment, LPS challenge was chosen as a representative, immune-based challenge that is known to activate the HPA axis through cytokine-mediated mechanisms. Male rats (*n* = 8 per group; *N* = 16) were given two cycles of intermittent ethanol exposure or vehicle intubations as described above. During adulthood (P73–75) rats were injected with 50 μg/kg (i.p.) LPS or equivolume vehicle. Blood was collected at baseline, 45, 90 and 180 min post injection.

### Experiment 3: Axis Reactivity to Ethanol Challenge Following Adolescent Ethanol Exposure

In the third experiment, rats were given an acute ethanol challenge during adulthood to test for durable changes in HPA axis sensitivity to the same challenge(s) utilized during adolescence, and to provide a referent to other published studies (Przybycien-Szymanska et al., 2010, 2011; Allen et al., 2016). Male rats (*n* = 8–10 per group; *N* = 18) were given two cycles of intermittent ethanol exposure or vehicle intubations as described above. During adulthood (P73–75), the test day began with collection of a baseline blood sample, after which rats were given a 2.5 g/kg i.p. ethanol or equivolume vehicle. Subsequent blood samples were collected 30, 60, 120 and 240 min post injection (see Figure 1A).

### Experiment 4: Sex-Differences in the Consequences of Adult LPS Challenge Following Adolescent Ethanol Exposure

The goals of the fourth experiment were to: (a) replicate the initial effect of reduced CORT and cytokine responses observed in adults with a history of adolescent alcohol exposure; (b) extend the period of alcohol exposure more broadly across the adolescent period; and (c) determine whether the impact of adolescent alcohol exposure on adult stress sensitivity would be sex-specific. Because early life shipping can produce variations in stress history and thereby influence stress reactivity (Laroche et al., 2009a,b; Vargas et al., 2016), rats for Experiment 4 were bred in house. Thus, in Experiment 4, rats (*n* = 6–8 per group; *N* = 30) were given four cycles of vehicle or ethanol intubations utilizing the procedure described above. After reaching adulthood, all rats received a 50 μg/kg i.p. LPS injection. Blood was collected at baseline, 45, 90, 180 and 360 min post injection.

## Results

### Experiments 1–3

#### Plasma Corticosterone Response to Challenges

Data were analyzed using a mixed between-subjects (adolescent Ethanol or Vehicle exposure) and repeated-measures (time course) analysis of variance (ANOVA). Restraint challenge showed significant CORT elevation peaking around 30 min and CORT levels returning to baseline by 120 min (*F*_(4,48)_ = 62.07, *p* < 0.001; Figure 1B). LPS challenge produced comparable increases in CORT across the 45 and 90 min time points, which then decreased by the 180 min time point (*F*_(3,42)_ = 22.64, *p* < 0.001; Figure 1C). Adult ethanol challenge increased CORT within 30 min and sustained high CORT levels through the 120 min time point (*F*_(4,64)_ = 70.65, *p* < 0.001). CORT still showed notable elevation at 240 min (Figure 1D), though the response was clearly resolving at this time point. Although no statistically significant effects of adolescent ethanol history on CORT response were observed on in response to the adult challenges, visual inspection of the data suggested a moderate attenuation of CORT in response to LPS challenge.

#### Blood Ethanol Concentrations Following Ethanol Challenge

As expected, rats injected with ethanol exhibited a marked and time-dependent elevation in BECs that peaked at 30–60 min and tapered over the remainder of the time course (*F*_(4,64)_ = 200.15, *p* < 0.001; Figure 1E). Rats with a history of adolescent alcohol exposure showed trended higher mean BECs at both 30 and 60 min after ethanol, though this effect was not statistically significant. It should be noted that mean peak BECs were approximately 25 mg/dL higher at both time points relative to rats that received vehicle during adolescence. Potential differences in alcohol pharmacokinetics in adolescent-exposed rats could therefore be an area worth examining in more targeted studies.

#### Blood Pellet mRNA Expression Levels Following Restraint Challenge

**Figure 2:**
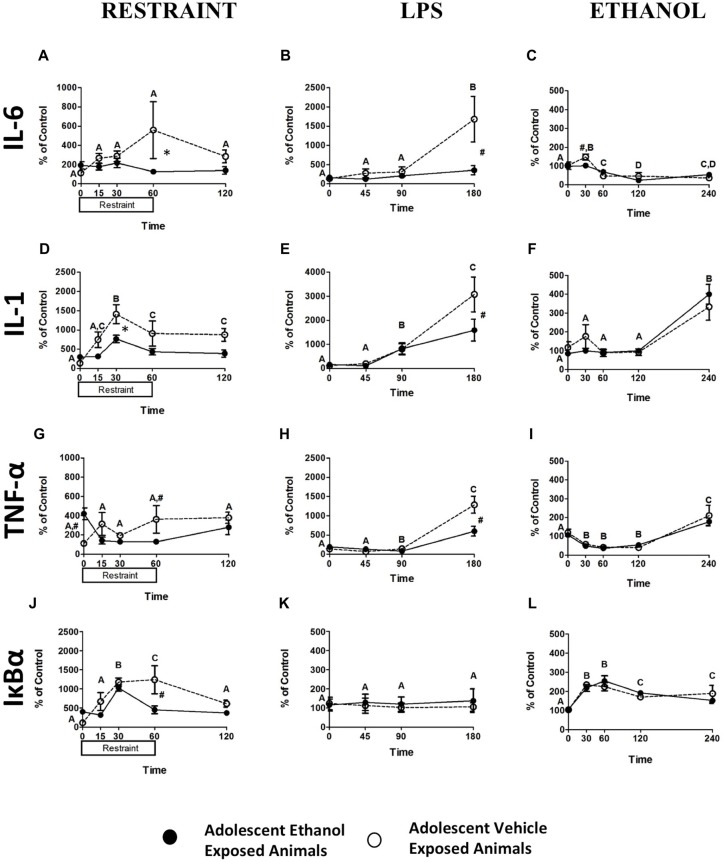
**Cytokine Expression from Experiments 1–3**. This figure shows mRNA expression changes following restraint, LPS and ethanol challenge in IL-6 (**A–C** respectively), IL-1 (**D–F** respectively), TNF-α (**G–I** respectively), IκBα (**J–L** respectively). Samples were collected at 0, 15, 30, 60 and 120 min following restraint, 0, 45, 90 and 180 min following LPS challenge, and 0, 30, 60, 120 and 240 min following ethanol challenge. Data is expressed relative to ultimate control (vehicle animals at the baseline time point) normalized to β-Actin. A significant main effect of time point is denoted by a lettering system in which time points that differ in letter are significantly different. A pound sign indicates a significant interaction between adolescent exposure and challenge stimulus at a given time point. An asterisk indicates a trend interaction between adolescent exposure and challenge stimulus at a given time point. All *post hoc* comparisons were made using Fisher’s Least Significant Difference *post hoc* test (*p* < 0.05).

While restraint challenge produced no significant alterations in IL-6 expression (Figure 2A), significantly increased IL-1β expression was noted at 30 min (*F*_(4,48)_ = 10.16, *p* < 0.001) and IL-1β remained elevated throughout the remainder of the time course (Figure 2D). No alterations in TNF-α expression were noted (Figure 2G) however; significant increases in Nuclear factor of kappa light polypeptide gene enhancer in B-cells inhibitor, alpha (IκBα) expression at 30 and 60 min (*F*_(4,48)_ = 13.53, *p* < 0.001) were seen that had returned to baseline levels by the 120 min time point (Figure 2J). A pattern showing that adolescent exposure to ethanol attenuated response to adult restraint challenge was seen in IL-6, IL-1β, TNF- α, and IκBα expression. A trend showing attenuation of IL-6 expression in the adolescent ethanol exposed rats was seen at the 60-min time point (*F*_(4,48)_ = 2.55, *p* = 0.051; Figure 2A). A similar trend showed that IL-1β expression was attenuated in the adolescent ethanol exposed rats at the 30-min time point (*F*_(4,48)_ = 2.44, *p* = 0.06; Figure 2D). Finally, a similar reduction was seen at the 60-min time point in both TNF-α (*F*_(4,48)_ = 5.49, *p* < 0.005; Figure 2G) and IκBα (*F*_(4,48)_ = 4.77, *p* < 0.005; Figure 2J) expression.

#### Blood Pellet mRNA Expression Levels Following LPS Challenge

Blood analysis revealed significant changes in expression of IL-6, IL-1β and TNF-α following LPS challenge. An increase in IL-6 expression at 180 min (*F*_(3,42)_ = 9.33, *p* < 0.001) as well as an overall decrease in IL-6 expression in rats exposed to ethanol in adolescence (*F*_(1,14)_ = 4.96, *p* < 0.05) was noted (Figure 2B). A distinct increase in IL-1β expression (*F*_(3,42)_ = 23.94, *p* < 0.001) occurred at 90 min and appeared to still be escalating at 180 min (Figure 2E). A similar pattern of results was found for TNF-α expression (*F*_(3,42)_ = 56.27, *p* < 0.001) with a profound increase occurring at 180 min and a decrease in overall expression in the adolescent exposed animals (*F*_(1,14)_ = 6.09, *p* < 0.05; Figure 2H). It was also found that adolescent ethanol exposure resulted in significant attenuation of IL-6 (*F*_(3,42)_ = 5.55, *p* < 0.05; Figure 2B), IL-1β (*F*_(3,42)_ = 3.07, *p* < 0.05; Figure 2E), and TNF-α (*F*_(3,42)_ = 10.60, *p* < 0.001; Figure 2H) expression at the 180 min time point in contrast to vehicle-exposed counterparts. Neither alterations in IκBα expression resultant from LPS challenge nor any significant effects of AIE were noted (Figure 2K).

#### Blood Pellet mRNA Expression Levels Following Ethanol Challenge

Data were analyzed using a mixed between subjects (adolescent Ethanol or Vehicle exposure) and repeated measures (0, 30, 60, 120 and 240 min post-injection) ANOVA. Analysis revealed significant changes in overall IL-6, IL-1β, TNF-α and IκBα expression across time. A significant decrease in overall IL-6 expression (*F*_(4,64)_ = 21.89, *p* < 0.001) was noted from 60 min post-challenge to 240 min (Figure 2C). Significant increases in IL-1β expression did not begin to occur until after 120 min showing continued escalation at the 240 min time point (*F*_(4,64)_ = 27.40, *p* < 0.001; Figure 2F). TNF-α also showed a significant reduction in expression (*F*_(4,64)_ = 39.58, *p* < 0.001) during the 30, 60 and 120 min time points and began to show rebound elevation at 240 min (Figure 2I). IκBα showed an increase in expression through the 60 min time point that had begun to taper by the 240 min time point (*F*_(4,64)_ = 17.27, *p* < 0.001; Figure 2L). Rats that received ethanol exposure during adolescence demonstrated significantly reduced IL-6 expression in contrast to vehicle-exposed rats (*F*_(4,64)_ = 3.03, *p* < 0.05; Figure 2C). No effects of adolescent ethanol exposure on IL-1β, TNF-α, or IκBα were observed.

### Experiment 4 Results

#### Corticosterone Response to Challenge

**Figure 3:**
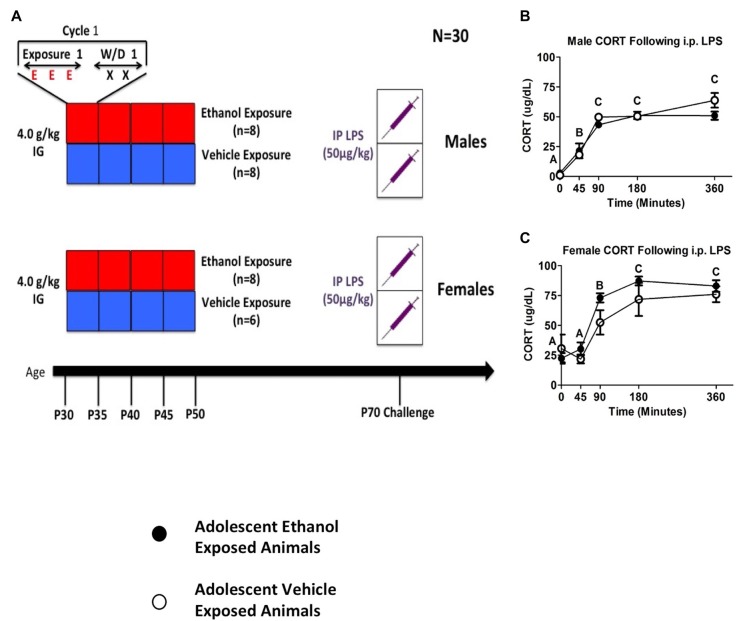
**Plasma CORT from Experiment 4**. The figure shows a schematic diagramming Experiment 4 **(A)**. It also shows plasma CORT levels in male and female rodents following 50 μg/kg i.p. LPS injection at 0, 45, 90, 180 and 360 min time points **(B, C)**. A significant main effect of time point is denoted by a lettering system in which time points that differ in letter are significantly different.

Data were analyzed using a mixed between-subjects (adolescent Ethanol or Vehicle exposure; male or female) and repeated-measures (time course) ANOVA. Sex-specific analyses were then run using a mixed between subjects (adolescent Ethanol or Vehicle exposure) and repeated measures (time course) ANOVA. When ANOVAs for each sex were run separately, LPS challenge produced significant increases in both male and female CORT release with female animals showing overall higher levels of CORT than males. A significant main effect of time point was found in both females (*F*_(4,48)_ = 40.86, *p* < 0.001; Figure 3C) as well as in males (*F*_(4,56)_ = 22.58, *p* < 0.001; Figure 3B). CORT increased at the 45-min time point and remained at peak through the 360-min time point in both males (*F*_(4,56)_ = 22.58, *p* < 0.001; Figure 3B) and females (*F*_(4,48)_ = 40.86, *p* < 0.001; Figure 3C). Although there was a trend indicating that adolescent ethanol exposure increased CORT release following LPS challenge in female rats (*F*_(4,48)_ = 1.68, *p* = 0.17), no such effect was seen in male rats (Figures 3B,C). A planned comparison examining CORT in female adolescent ethanol and vehicle exposed animals at the 90 min timepoint revealed a significant sensitization of LPS induced CORT in adolescent ethanol exposed animals when compared to vehicle exposed animals at the same timepoint (*t*_(12)_ = 2.34, *p* < 0.05).

#### Blood Pellet mRNA Expression Levels Following LPS Challenge

**Figure 4:**
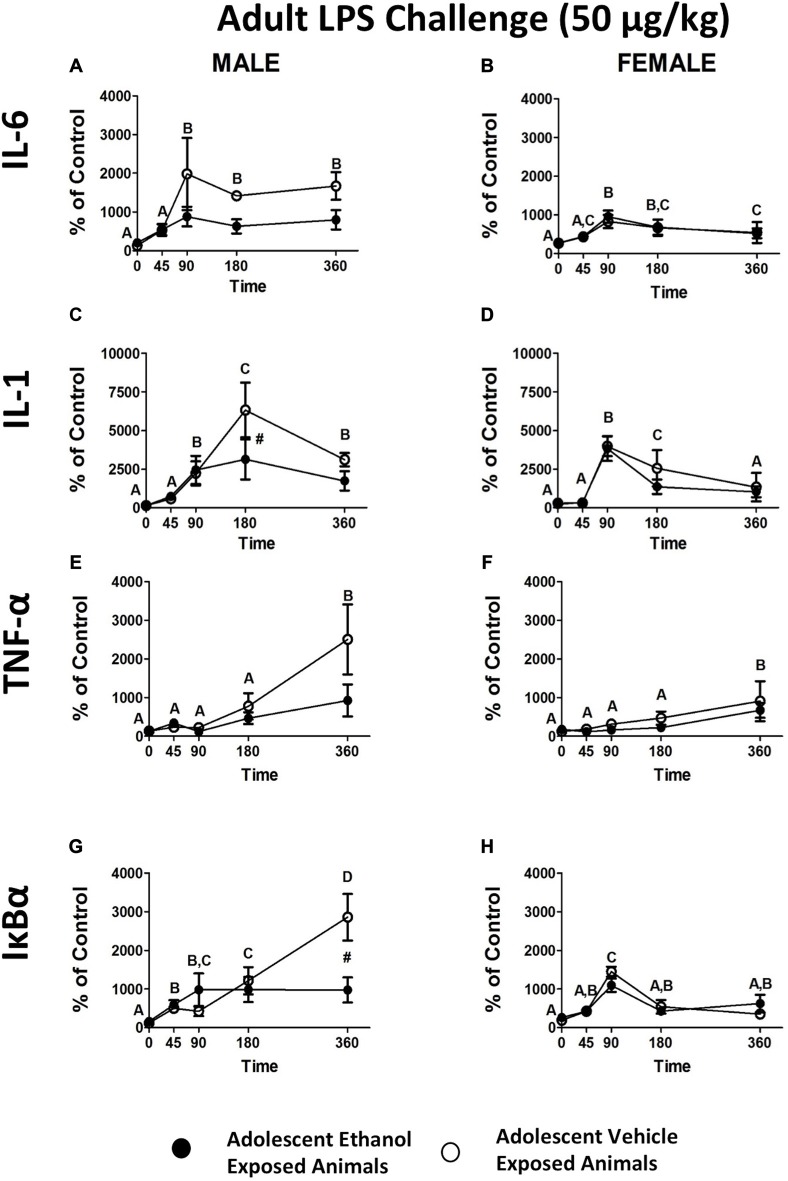
**Cytokine expression in male and female rats from experiment 4**. This figure shows mRNA expression changes in male and female rats following i.p. LPS injection in IL-6 **(A,B)**, IL-1 **(C,D)**, TNF-α **(E,F)** and IκBα **(G,H)**. Samples were collected at 0, 45, 90, 180 and 360 min following LPS injection. Data is expressed relative to ultimate control (Vehicle animals at the baseline time point) normalized to β-Actin. A significant main effect of time point is denoted by a lettering system in which time points that differ in letter are significantly different. A pound sign indicates a significant interaction between adolescent exposure and challenge stimulus at a given time point. All *post hoc* comparisons were made using Fisher’s Least Significant Difference *post hoc* test (*p* < 0.05).

When the data were analyzed using an omnibus ANOVA including sex as a variable, a significant sex by adolescent interaction was noted (*F*_(1,26)_ = 7.20, *p* < 0.05; Figures 4A,B). When ANOVAs for each sex were run separately, IL-6 showed a trend in the male adolescent ethanol exposed rats showing attenuation of expression in contrast to vehicle exposed animals (*F*_(4,56)_ = 2.04, *p* = 0.10; Figure 4A). No such effects were noted in female animals (Figure 4B). While adolescent ethanol exposure visibily reduced IL-6 expression in male rats it had little recognizable effect on IL-6 expression in the females.

When data were analyzed using an omnibus ANOVA including sex as a variable, rats that received adolescent ethanol exposure showed significantly reduced levels of IL-1β expression when compared to vehicle exposed counterparts (*F*_(4,104)_ = 2.95, *p* < 0.05) however, this appears to be largely a result of the differences noted in the male animals (Figures 4C,D). When ANOVAs for each sex were run separately, a significant attenuation of IL-1β expression was replicated in the male, adolescent ethanol exposed rats at the 180 min time point (*F*_(4,56)_ = 3.44, *p* < 0.05; Figure 4C). Interestingly, no such interaction was noted for the female animals although a significant increase in overall expression was noted at the 90 and 180 min time points (*F*_(4,48)_ = 14.63, *p* < 0.001; Figure 4D).

When data were analyzed using an omnibus ANOVA including sex as a variable, males showed much greater TNF-α increase in expression over the time course in contrast to female counterparts, regardless of adolescent exposure (*F*_(4,104)_ = 2.71, *p* < 0.05; Figures 4E,F). Regardless of sex, TNF-α expression increased with peak levels being reached by the 360 min time point (*F*_(4,104)_ = 11.06, *p* < 0.001; Figures 4E,F). When ANOVAs for each sex were run separately, TNF-α expression in males showed a similar pattern of expression as in the previous experiment with a noticeable but not significant attenuation of expression developing in the adolescent ethanol exposed animals. Males showed much higher overall levels of expression than females (*F*_(1,26)_ = 5.07, *p* < 0.05; Figures 4E,F). Females also showed a significant increase in overall TNF-α expression at the 360 min time point (*F*_(4,48)_ = 4.90765, *p* < 0.05; Figure 4F).

IκBα closely mirrored the pattern of expression observed in Experiment 1. When data were analyzed using an omnibus ANOVA including sex as a variable, a significant three way interaction between adolescent exposure, sex and treatment (*F*_(4,104)_ = 3.60, *p* < 0.01) revealed that again adolescent ethanol exposure resulted in significant attenuation of IκBα exposure but exclusively in the male animals (Figures 4G,H). Female animals showed little to no consequence of adolescent ethanol exposure. Interestingly while in both female and male rats IκBα expression peaked at approximately the 1500% mark in the vehicle exposed male rats expression continued to escalate until the 360 min time point reaching almost double the magnitude of expression seen in the remaining three groups (Figures 4G,H).

## Discussion

Our primary goal in these studies was to examine how AIE would alter the HPA axis response to different types of challenge in adulthood. This set of studies shows that not only does adolescent ethanol exposure produce lasting changes in HPA axis sensitivity into adulthood, but that these changes are both sexually dimorphic and stimulus specific. Whereas adolescent ethanol exposed male rats showed blunting of peripheral cytokine expression in several key pro-inflammatory cytokines in response to restraint and LPS administered during adulthood, minimal differences were observed in response to ethanol challenge. These findings suggest that the HPA axis is not debilitated by adolescent ethanol challenge and might suggest adaptations that occur extrinsic to the axis. The three challenges used in the study each represent unique modalities for stimulating the HPA axis, restraint being psychological, LPS being immune, and ethanol being the same stimulus used to invoke the changes. Each stimulus produced a unique profile of cytokine expression in blood and the effects of AIE were different between challenge modality.

Alcohol is known to produce immune dysfunction. However, unlike other illnesses, the immune dysfunction that occurs is rarely at a level that would be considered clinical, rather it becomes notable following exposure to a subsequent challenge (Szabo and Saha, 2015). One example of this is the increased susceptibility to bacterial infections such as pneumonia and tuberculosis that occurs in heavy drinkers and alcohol dependent individuals (Cook, 1998; Ogunsakin et al., 2016). In addition, evidence from both human and animal studies has shown that prenatal ethanol exposure can result in increased vulnerability to infection and disease that may persist across the lifespan (Gauthier, 2015). In this study, a pronounced immunosuppressive effect of AIE in response to subsequent LPS challenge was seen in male rats while no difference was noted in females. In contrast, no effect of AIE on the CORT response in male rats was noted, yet a sensitization was observed in females. Although the functional significance for males and females with a history of adolescent ethanol exposure remains unclear, these divergent outcomes suggest that males may be more prone to infection, whereas females may be more prone to stress-related disorders (depression, anxiety) that are often co-morbid with HPA axis dysregulation (Weinberg et al., 2008). In this way, adolescent ethanol exposure may contribute to sex differences in specific disease vulnerabilities that emerge later in life.

Prenatal Alcohol Exposure (PAE) has also been shown to alter HPA axis reactivity in adulthood, suggesting there may be multiple developmental windows during which ethanol can impact later stress sensitivity. For instance, PAE-exposed animals show hyperactivity of the CORT response to restraint stress imposed later in life (Weinberg, 1992; Wieczorek et al., 2015). This hyperactivity has also been shown in response to LPS challenge, IL-1β challenge, local inflammatory challenge probed via turpentine injection, and other types of stressors (Lee and Rivier, 1996; Raineki et al., 2014). The consequences of PAE on adult challenge also appear to be sexually dimorphic as well as stimulus specific. PAE has led to an enhanced HPA axis response in adults to stressors such as acute restraint and forced swim in female rats but not in males (Weinberg et al., 2008). In contrast, solely PAE-exposed male rodents have shown a hyper-reactive HPA response to prolonged restraint and exposure to cold (Kim et al., 1999; Weinberg et al., 2008). Both sexes have shown hyper-reactivity of the axis to immune challenges such as LPS and IL-1β challenge (Weinberg et al., 2008). The results of the present studies clearly demonstrate that adolescent ethanol exposure is capable of causing changes in HPA axis reactivity lasting into adulthood. Based on the results of this study, the effects of adolescent ethanol exposure on later adult challenge appear to be stimulus specific and show sex specific effects, much like has been shown in studies involving PAE.

Prior work from our lab demonstrated that male adolescent rats displayed a blunted cytokine response in the CNS following either LPS challenge or ethanol challenge relative to adult comparators (Doremus-Fitzwater et al., 2015). One potential interpretation of the blunted cytokine responses observed in adolescent-exposed males in the present studies is that this may represent a “locking-in-like” effect. It is noteworthy, however, that, circulating concentrations of endotoxin following LPS challenge were substantially reduced in adolescents compared to adults, suggesting that adolescents may not process LPS in the same way as their adult counterparts. Whether this effect is through differential uptake and transit of LPS into the bloodstream, enhanced clearance of LPS from blood, or other immunological differences between adolescents and adults remains unclear. Nevertheless, plasma endotoxin concentrations were predictive of the HPA axis response (Doremus-Fitzwater et al., 2015). Unfortunately, it was not possible to measure plasma endotoxin concentrations in the present studies due to the limited available sample. Future studies will be necessary to test whether adolescent alcohol exposure impacts subsequent immune processing of LPS in a manner consistent with a “locking in-like” effect. The lack of AIE effects on cytokine expression in female rodents could suggest that female adolescent and adults would not demonstrate the previously noted differences in response to LPS challenge or that females displayed some form of resiliency to the changes that resulted in the “locking-in-like” effect.

Future studies should further probe the mechanisms by which adolescent alcohol exposure influences adult cytokine reactivity and stress sensitivity. LPS invokes an inflammatory response at least in part through its effects on Toll-like receptor 4 (TLR4), activating the NFKB pathway, and ultimately stimulating inflammatory cytokine expression and release (Doremus-Fitzwater et al., 2015). Female rodents have shown an enhanced ethanol-induced inflammatory response during adolescence, as exhibited by increased inflammatory cytokine levels in serum and in brain, however, this effect was not observed in male rats (Pascual et al., 2016). This difference may be mediated by up-regulated TLR4 expression during intoxication that are more pronounced in female rodents than in males, as supported by the fact that TLR4 KO mice do not exhibit these cytokine differences (Pascual et al., 2016). Binge ethanol expression has also been shown to interfere with LPS induced NFKB activity. Ethanol induces Heat shock factor protein 1 (HSF1) and 70 kilodalton heat shock protein (hsp70), two enzymes that ultimately inhibit TLR4/Myeloid differentiation primary response gene (MYD88) signaling via inhibition of the NFKB pathway (Muralidharan et al., 2014). This results in something akin to endotoxin tolerance in monocytes and macrophages pretreated with ethanol (Muralidharan et al., 2014). Lingering inhibition of TLR4/MYD88 signaling resulting from binge ethanol exposure could lead to a reduced cytokine response in male rats that could be normalized in females through sensitized TLR4 expression in female rats following AIE. While this is just one potential explanation for the effects seen in this study, future studies should address the mechanism of these changes and TLR4 signaling pathways might be a good place to start.

Although the results of this study showed few consequences of adolescent ethanol exposure on the response to adult ethanol challenge, the existing literature examining similar manipulations is conflicting. The work of Pak using a similar model of ethanol administration has shown sensitization of the axis response in males following adult ethanol challenge (Przybycien-Szymanska et al., 2010, 2011). In contrast, a different study in which male adolescent rats received AIE and were challenged with ethanol in adulthood showed blunting of the axis response to later ethanol challenge (Allen et al., 2016). There are a number of factors that could account for this disparity. In the first set of studies, the number of ethanol cycles as well as the challenge dose was different, and the experiments were conducted using Wistar rats. In the second set of studies the method of adolescent administration was via daily vapor inhalation, whereas our studies utilized an intermittent, intragastric administration procedure. This could suggest that the dose, route, and schedule of ethanol exposure during adolescence may be critical determinants of long-term changes in HPA axis reactivity and cytokine gene induction.

One important question to address in future studies will be the mechanisms underlying the apparent HPA axis sensitization observed in females as a result of adolescent ethanol exposure. In particular, it will be important to identify whether these changes reflect altered function of neural circuits that govern HPA axis regulation, or intrinsic differences in sensitivity of endocrine glands comprising the HPA axis. This can be readily tested through a systematic series of hormonal challenges (CRH or ACTH injection) as we have done under other circumstances (Hueston and Deak, 2014b; also see Spencer and Deak, 2016 for an overview). Such studies are planned for the near future. It will also be important to extend these physiological findings to behavioral circumstances as well. For instance, the suppressed cytokine response evoked by LPS would predict that males with a history of adolescent ethanol exposure should display reduced sickness behavior under conditions of acute illness as adults. Given the HPA dysregulation observed in females with an adolescent history of ethanol, one might predict heightened anxiety and/or depression in adulthood. Given the established association between age of first alcohol use and subsequent vulnerability to addiction (Ystrom et al., 2014), it will also be important to assess whether adolescent ethanol exposure might increase vulnerability to addiction, particularly in females since stress dysregulation has been posited as one pathway towards addiction (Miller and Spear, 2006; Koob, 2008). These and many other issues will need to be explored in future studies.

As with any pre-clinical model of ethanol exposure, the mode of ethanol delivery and subsequent BECs achieved require careful consideration. We elected to utilize intragastric intubation for these studies because it mimics the normal route of administration for ethanol, and allows for binge-like doses to be achieved consistently and reliably. Such BECs would be difficult to achieve through voluntary consumption in rat, though that may be a path for future studies. BECs during the adolescent intubation regimen were not assessed due to concerns that the blood sampling procedure might have an enduring influence on the study outcomes. However, prior studies from our lab reported peak BECs of approximately 180 mg/dl after delivery of 4 g/kg (i.g.) of ethanol, which is consistent with a binge-like range of BECs (Buck et al., 2011; Doremus-Fitzwater et al., 2015). Because BECs were not assessed in the present studies, we cannot rule out the possibility that the sex-specific outcomes observed here might be due to intrinsic differences in ethanol metabolism (or other pharmacokinetic differences) between males and females (Truxell et al., 2007). Finally, our studies did not include a group of non-intubated controls to assess the influence of the intubation procedure itself. We occasionally use non-intubated controls and have not seen any evidence emerge to demonstrate long-term effects of the intubation procedure itself thus far. For practical purposes, however, we cannot do so in every study design. Nevertheless, these issues raise a more general question regarding whether any stress challenge (not just ethanol) imposed during adolescence might lead to similar outcomes. This is an interesting question that will have to be resolved in future studies. Overall, any ethanol delivery approach has both strengths and limitations and the study outcomes should be considered within an appropriate framework.

While this study yielded several interesting results, there are several procedural differences that must be considered in the interpretation of study outcomes. For instance, Experiments 1–3 utilize rats shipped from a vendor post weaning, whereas Experiment 4 utilized rats bred in house. The use of shipped animals for developmental studies is not ideal (see Laroche et al., 2009a,b; Spencer and Deak, 2016; Vargas et al., 2016), though it remains common practice for many investigators who do not have access to a breeding facility. Despite this limitation, Experiments 2 and 4 both yielded similar, durable influences of adolescent ethanol on LPS-induced cytokines as adults, even though the overall magnitude of cytokine responses across studies varied widely. A second disparity in study design was the number of ethanol cycles that the animals received during adolescence. Two cycles of intermittent ethanol exposure were utilized in Experiments 1–3, whereas four cycles were utilized in Experiment 4. Ultimately, the increase in number of cycles did not alter the directionality of the effects seen although it did appear to increase their magnitude. The model of ethanol administration is an important consideration because other studies have suggested that it is not only the amount of ethanol that is the dominant factor in creating durable effects resulting from AIE, but also the number of periods of abstinence between ethanol exposures (Lopez and Becker, 2005; Diaz-Granados and Graham, 2007). Indeed, periods of acute withdrawal that occur throughout intermittent ethanol exposure may play a critical role in causing some of the lasting changes of ethanol (Spear, 2016). Finally, to truly attribute the results of this study to an adolescent critical period, follow up studies that incorporate an adult control (that receive the same quantity of ethanol over the same time frame) and that control for the time difference between AIE and challenge between the two studies would be needed. Despite these minor limitations, the present study yielded important and highly consistent results that warrant further investigation.

A final element of the study that affects interpretation of the results is the use of the cellular fraction of blood to study peripheral cytokine expression (Wang et al., 2016; Opstad et al., 2017). Although it is more common in contemporary studies to utilize plasma protein measures to detect cytokines, cytokines in plasma have very short half-lives and the assays to measure them require comparatively large samples. Thus, a viable alternative for procuring cytokine signals is to utilize gene expression in the cellular fraction, which can be done in very small samples, thereby allowing for the use of a within-subjects experimental design. Although there is always a question as to how such gene expression changes might align with functional protein measures, the detailed time course information gathered in the present study can be used to inform selection of more discrete time points for protein measures. Such studies are already planned in the near future. Furthermore, while the functional relationship between peripheral cytokines and central cytokines is unclear, prior studies have suggested that peripheral cytokine release may play a role in activating subsequent central action. LPS induced TNF-α in serum has been shown to play a role in triggering a proinflammatory response in the brain (Qin et al., 2007, 2008). Further research probing the relationship between these elements of the immune response would be worthwhile.

In conclusion, the effects of ethanol during adolescence appear to be mitigated by many factors. The results of this study as well as other work suggest that how the ethanol is administered, the nature/modality of the subsequent challenge stimulus, and the sex of the animals all may interact with AIE. While the rates of binge drinking in both adolescents and adults is very high, men are almost twice as likely to engage in binge drinking as women (Centers for Disease Control and Prevention, 2012). Differences in how men and women may be affected by ethanol, particularly during periods of developmental vulnerability, may contribute to the difference in AUD prevalence that exists between men and women (Nolen-Hoeksema, 2004). Despite all of this, it has been reported that women suffer more negative consequences of heavy ethanol use, including increased susceptibility to physical illness at lower levels of exposure than has been seen in men (Nolen-Hoeksema, 2004). Several drugs of abuse have been linked to weakened natural immune system function during use and at times lingering afterwards. Ethanol abuse specifically has been shown to produce a pronounced immunosuppressive effect and can increase the chances of individuals with ethanol being vulnerable to infection (Szabo, 1997; Szabo and Saha, 2015). Alterations in HPA axis reactivity and cytokine function could help to explain some of ethanol’s effects on the immune system and the potential for adolescent ethanol use to cause lasting alterations in immune function. This could lead to a lifetime of heightened immune vulnerability to an array of types of challenge stimuli. Better understanding of how adolescent ethanol exposure affects the immune system and the longevity of such changes could help to better understand the risk of individuals exposed to future insults and help identify targets to reverse negative alterations.

## Author Contributions

ASV, TD-F, AG and TD made substantial contributions to the conception or design of the work as well as participated in the acquisition, analysis, or interpretation of data for the work. In addition, they played a significant role in drafting the work or revising it critically for important intellectual content and submitted for final approval of the version to be published. All authors agree to be accountable for all aspects of the work in ensuring that questions related to the accuracy or integrity of any part of the work are appropriately investigated and resolved.

## Funding

Research reported in this publication was supported by the National Institute on Alcohol Abuse and Alcoholism of the National Institute of Health under Award Number P50AA017823 to TD and the Center for Development and Behavioral Neuroscience at Binghamton University. Any opinions, findings and conclusions or recommendations expressed in this material are those of the author(s) and do not necessarily reflect the views of the above stated funding agencies.

## Conflict of Interest Statement

The authors declare that the research was conducted in the absence of any commercial or financial relationships that could be construed as a potential conflict of interest.

## Abbreviations

ACTH: Adrenocorticotropic hormone
AIE: Adolescent intermittent ethanol
ANOVA: Analysis of variance
AUD: Alcohol use disorder
AVP: Vasopressin
BALs: Blood alcohol levels
BECs: Blood ethanol concentrations
C: Celcius
CORT: Corticosterone
CRH: Corticotropin-releasing hormone
CTA: Conditioned taste aversion
HPA: Hypothalamic-Pituitary-Adrenal
HSF1: Heat shock factor protein 1
Hsp70: 70 kilodalton heat shock protein
IACUC: Institutional animal care and use committee
i.g.: Intragastric
IκBα: Nuclear factor of kappa light polypeptide gene enhancer in B-cells inhibitor, alpha
IL: Interleukin
i.p.: Intraperitoneal
LPS: Lipopolysaccharide
MYD88: Myeloid differentiation primary response gene
NFκB: Nuclear factor kappa-light-chain-enhancer of activated B cells
P: Postnatal day
PAE: Prenatal alcohol exposure
PVN: Paraventricular nucleus of the hypothalamus
RT-PCR: Real time reverse transcription polymerase chain reaction
TLR4: Toll-like receptor 4.
